# Impact of Frozen and Conventional Elephant Trunk on Aortic New-Onset Thrombus and Inflammatory Response

**DOI:** 10.3390/diagnostics12102511

**Published:** 2022-10-17

**Authors:** Elena Marchiori, Alexander Oberhuber, Sven Martens, Andreas Rukosujew, Abdulhakim Ibrahim

**Affiliations:** 1 Department of Vascular and Endovascular Surgery, University Hospital Muenster, 48149 Muenster, Germany; 2 Department of Cardiothoracic Surgery, Division of Cardiac Surgery, University Hospital Muenster, 48149 Muenster, Germany

**Keywords:** post-implantation syndrome, frozen elephant trunk, conventional elephant trunk, aortic dissection, aortic aneurysm

## Abstract

(1) Aim: The primary endpoint of this study was to evaluate the impact of frozen elephant trunk (FET) and conventional elephant trunk (CET) on aortic mural thrombus. The secondary endpoint was to investigate the incidence of persistent inflammatory response (IR) in the form of post-implantation syndrome (PIS) or persistent fever without infection focus after FET and CET, respectively, as well as the risk factors associated with its occurrence. (2) Methods: A single-center, retrospective, observational study of 57 consecutive patients treated with FET and CET between April 2015 and June 2020 was performed. Demographics, procedural data, perioperative laboratory exams as well as vital parameters were recorded. Pre- and postoperative computer tomography angiography (CTA) scans were analyzed with a dedicated software. IR was defined as the presence of continuous fever (>38°, lasting > 24 h) and leukocytosis (white blood cell count > 12 × 1000/µL) developing after surgery in the absence of an infection focus. (3) Results: Fifty-seven consecutive patients (mean age 58.4 ± 12.6 years, 36.8% females) treated with FET (66.6%) or CET (33.3%) for acute aortic dissection (56.1%), post-dissection-aneurysm (19.2%) or aortic aneurysm (24.5%) were included. The median thrombus volume on CTA preoperatively was 10.1 cm^3^ (range 2–408 cm^3^). After surgery, the median new-onset mural thrombus was 9.7 cm^3^ (range 0.2–376 cm^3^). Nineteen (33.3%) patients developed IR; patients with IR were significantly younger (*p* = 0.027), less frequently of female gender (*p* = 0.003) and more frequently affected from acute dissection (*p* = 0.002) and stayed in the intensive care unit (ICU) significantly longer (*p* = 0.033) than those without IR. Postoperatively, the volume of new-onset thrombus was significantly greater in the IR group (84.4 vs. 3.2 cm^3^, *p* < 0.001). (4) Conclusions: In the context of CET and FET, the persistent inflammatory response occurred in 33.3% of the patients with persistent fever without infection focus. IR was associated with a higher volume of new-onset thrombus and significantly prolonged ICU stay. Further studies to investigate these observations are needed.

## 1. Introduction

Conventional and frozen elephant trunks are well-established procedures for the treatment of extensive aortic pathologies [1,2]. The conventional elephant trunk (CET) procedure was first described by Borst et al. [3] in 1983 and relies on a tube arch prosthesis with a free-floating extension in the proximal descending aorta. The frozen elephant trunk (FET), introduced in 2003 [4], is based on a hybrid prosthesis consisting proximally in a conventional Dacron tube and distally in a covered stent graft, thus combining open treatment for the aortic arch and endovascular repair for the descending thoracic aorta in one step.

Systemic stress response and inflammatory activation are known to occur during cardiac surgery such as CET and FET. The persistent inflammatory response (IR) consisting of continuous fever without infection focus postoperatively can affect the recovery of these patients. In open surgery, the inflammatory response is classically triggered by surgical trauma due to incision, dissections and tissue manipulation; in the setting of endovascular stent graft implantation, a prolonged inflammatory response can be driven by a sterile inflammation called post-implantation syndrome (PIS). 

In the perioperative care of endovascular-treated patients, post-implantation syndrome is a well-known clinical entity. It was initially described, after endovascular aneurysm repair (EVAR) for abdominal aortic aneurysm (AAA) [5], as a systemic inflammatory response occurring in the early postoperative period and is defined as continuous fever and leukocytosis in the absence of an infection focus; its incidence has been reported amounting to circa 45% in the context of FET [6]. With the growing application of hybrid stent grafts such as FET for aortic arch lesions and for extensive thoracoabdominal aortic pathologies, PIS issues are becoming increasing relevant in postoperative care. Based on the current evidence in the context of endovascular aneurysm repair, PIS incidence may be triggered by the formation of new-onset mural thrombus [7,8,9]. The impact of PIS on patients’ outcome is still controversial, with some studies reporting, however, a significantly prolonged hospitalization [10] and increasing rates of major adverse events at follow-up [11].

The primary endpoint of this study was to evaluate the impact of frozen and conventional elephant trunks on the onset of aortic mural thrombus. The secondary endpoint was to investigate the incidence of persistent inflammatory response (IR) after CET/FET and examine risk factors associate with its occurrence.

## 2. Materials and Methods

This was a retrospective, single-center, observational study including patients who underwent CET or FET between April 2015 and June 2020. The study was approved by the local ethics committee (protocol number 2018-506-f-S).

Exclusion criteria were history of previous surgery; malignancy; anti-inflammatory or glucocorticoid treatment at admission; preoperative evidence of infection; reoperations (inclusive TEVAR), or death within seven postoperative days; postoperative clinical or instrumental evidence of infection; incomplete chemical laboratory or microbiological data. Patient demographics, comorbidities, preoperative medications, laboratory and microbiological findings, vital parameters as well as perioperative radiological imaging were collected.

Laboratory analyses, including white blood cell count (WBCC), C-reactive protein (CRP) and procalcitonin (PCT), were performed in all patients before surgery and at least daily in the seven days thereafter; by multiple records, the highest daily value was used. Diagnostics in the suspect of a focus of infection is well-standardized at our institution, and microbiology cultures consisting in the analysis of urine, sputum, stool as well as blood cultures are routinely performed in the presence of fever or elevated laboratory parameters; in all patients, the presence and findings of microbiology cultures were checked. Body temperature measured preferentially as bladder temperature, alternatively as tympanic temperature, was recorded every eight hours during the entire duration of the hospitalization. Pre- and postoperative computer tomography angiography (CTA) scans were collected and analyzed with a dedicated software (Aquarius iNtuition, TeraRecon, Foster City, CA, USA). IR and PIS were defined as the presence of continuous fever (>38°, lasting longer than 24 h) and leukocytosis (white blood cell count > 12 × 1000/µL) with development after surgery in the absence of infection and in the existence of negative microbiology cultures. From initially 101 patients, a total number of 44 patients were excluded because of clinical and/or laboratory signs of recent infection (positive blood and/or urine culture, pathological chest-X-ray as a sign for pneumonia), treatment with glucocorticoid drugs, malignant tumor and patient with surgical revision during the first postoperative week. Finally, 57 patients were included in the study.

### 2.1. Surgical Procedure

The detailed surgical techniques of CET and FET were reported previously [12,13]. 

In summary, both CET and FET procedures were performed through 1a median sternotomy under moderate hypothermic circulatory arrest at 28 °C and with selective antegrade cerebral perfusion. Extracorporeal circulation was initiated immediately after median sternotomy, and myocardial protection was performed with retrograde cold blood cardioplegia. For FET patients, the Thoraflex™ Hybrid Plexus Prosthesis (Vascutek, Terumo Aortic, Inchinnan, Scotland, UK) was routinely used; for CET, a multibranched prosthesis (Vascutek, Terumo Aortic, Inchinnan, Scotland, UK) was used. After completion of the distal anastomosis of CET or FET, the perfusion of the lower body was reinitialized, and the aortic arch vessel was anastomosed and reconstructed. All FET patients were treated by the same cardiac surgeon; CET patients were treated by two different operators.

### 2.2. Thrombus Volume Rendering and Measurements

**Figure 1:**
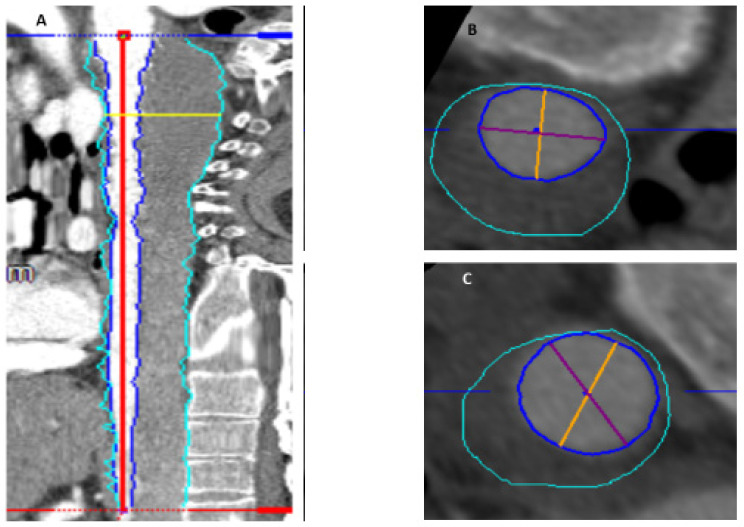
Thrombus volume measurements before and after the FET/CET procedure using a dedicated software. (**A**) Centerline in the true lumen, thrombus area has been marked and measured manually for each CT slice (grey area), separated from the true lumen. (**B**,**C**) Volume of the thrombus was measured by subtracting the true lumen from total aortic diameter.

Thrombus volume calculations of the aortic segment from the left subclavian artery until the celiac trunk were performed on preoperative and postoperative computer tomography angiography (CTA) scans. Three-dimensional CTA reconstruction was performed by means of a dedicated software (Aquarius iNtuition, TeraRecon, Foster City, CA, USA); the step-by step thrombus volume measurement technique was previously described [14]. Volume rendering and measurements were performed by two experienced vascular surgeons in order to reduce bias. Using multiplanar planar reconstruction (MPR) and curved planar reconstruction tools, the aortic lumen and thrombus volume were visualized in the venous phase. After generation of the centerline, the thrombus areas were multiplanarily contoured and displayed in straight MPR reconstruction, and the marked areas were checked and manually corrected whenever necessary. In the preoperative CTA after contouring and selection, the chronic mural thrombus was quantified using the volume calculator function. Slices of the postoperative CTA were analogously reconstructed and marked, and the new-onset thrombus volume was calculated as the difference between the postoperative total thrombus volume and the volume obtained in the preoperative CTA (Figure 1).

### 2.3. Endpoints

The primary endpoint of the study was the assessment of the thrombus volume and the incidence of IR in the CET/FET cohort. The secondary endpoint was to investigate the predisposition factor for IR occurrence, including anamnestic, comorbidity, procedure-related and postoperative risk factors.

### 2.4. Statistical Analyses

Statistical analyses were performed using SPSS software version 26.0 (IBM Corp., Armonk, NY, USA). For parametric data, continuous variables were expressed as mean ± standard deviation, for non-parametric data as median with interquartile range and dichotomous variables were presented as numbers and percentages. Comparisons were performed using a Student’s t-test for continuous normally distributed variables and using a Mann–Whitney U-test for non-normally distributed variables. For categorical variables, a chi-square test or Fisher’s exact test was used. Significant variables were then included in a multivariate regression analysis. Multivariate logistic regression was interpreted with an odds ratio, 95% confidence interval and *p*-value. The threshold of statistical significance was set to a value of *p* < 0.05.

## 3. Results

The study included fifty-seven consecutive patients. Thirty-eight patients (66.6%) underwent aortic replacement with the FET technique using a Thoraflex™ Hybrid Plexus Prosthesis (Vascutek, Terumo Aortic, Inchinnan, Scotland, UK), and nineteen patients (33.3%) with the CET procedure with a multibranched prosthesis (Vascutek, Terumo Aortic, Inchinnan, Scotland, UK). The mean age was 58.4 ± 12.6 years, and 36.8% of the patients (n = 21) were females. In total, 32 patients (56.1%) had an acute aortic dissection, 11 patients (19.2%) a post-dissection aneurysm and 14 patients (24.5%) had an aortic aneurysm without dissection. The mean maximum diameter of the aneurysm was 65.7 ± 2.5 mm. The most common comorbidity was hypertension (78.9%, n = 45), followed by cardiac pathologies such as chronic heart disease, coronary artery disease, atrial fibrillation (17.5%, n = 10; 15.7%, n = 9; and 15.7%, n = 9, respectively) and chronic obstructive pulmonary disease (14%, n = 8). Twenty-four patients (42.1%) were past or active smokers. Regarding medical therapy, the most common medications were β-blockers (54.3%, n = 31), followed by angiotensin-converting enzyme inhibitors (26.3%, n = 15), statins (17.5%, n = 10) and aspirin (10.5%, n = 6). Patients’ baseline data, demographics and comorbidities are summarized in Table 1.

Regarding procedural and perioperative data, the mean duration of the operation was 336.4 ± 76.4 min, while the mean intensive care unit stay was 7.2 ± 5.3 days. The average in-hospital length of stay was 14.7 ± 5.9 days. Thirty-eight patients (66.6%) underwent aortic replacement with FET, and nineteen patients (33.3%) underwent CET. The median thrombus volume on computed tomography angiograms preoperatively was 10.1 cm^3^ (range 2–408 cm^3^). After surgery, the median new-onset mural thrombus was 9.7 cm^3^ (range 0.2–376 cm^3^) (Table 2). 

**Figure 2:**
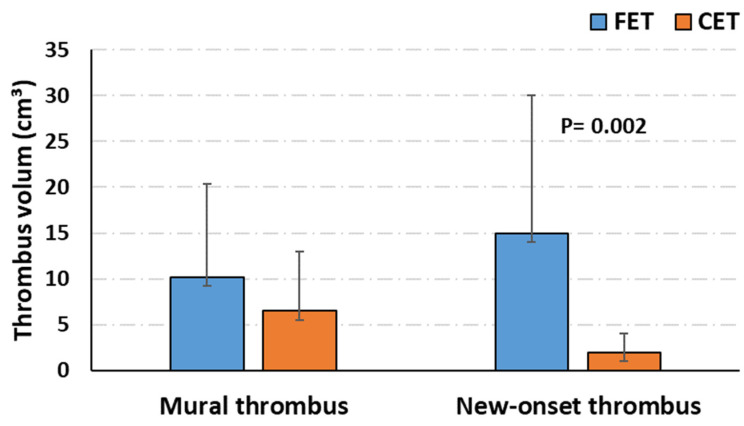
Box plot representing preoperative and postoperative thrombus volume.

The occurrence of IR was observed in nineteen (33.3%) patients. In terms of baseline characteristics, patients with IR were significantly younger (53.8 ± 8.9 vs. 60.7 ± 9.6 years, *p* = 0.027), less frequently of female gender (3.5% vs. 33.3%, *p* = 0.003) and more frequently suffered from acute dissection (*p* = 0.002) than those without IR. The duration of the operative procedures did not differ significantly in the two groups (mean duration 348.2 ± 95.6 vs. 330.6 ± 65.4 min, *p* = 0.418), whereas the intensive care unit stay was significantly longer in the IR group (9.5 ± 5.8 vs. 6.08 ± 4.7, *p* = 0.033). Patients undergoing the conventional elephant trunk were less likely to develop IR (10.5% vs. 89.4%, *p* = 0.009). Thrombus volume was not significantly different before the procedure (9.1 vs. 14 cm^3^, *p* = 0.224), and the volume of postoperative new-onset thrombus was significantly greater in the IR group (84.4 vs. 3.2 cm^3^, *p* < 0.001). Preoperatively, the chronic mural thrombus volume was comparable in the FET and CET group, while postoperatively, patients treated with FET exhibited a significantly greater new-onset thrombus volume compared with CET patients (Figure 2). Postoperative inflammatory markers are summarized in Table 3. 

**Figure 3:**
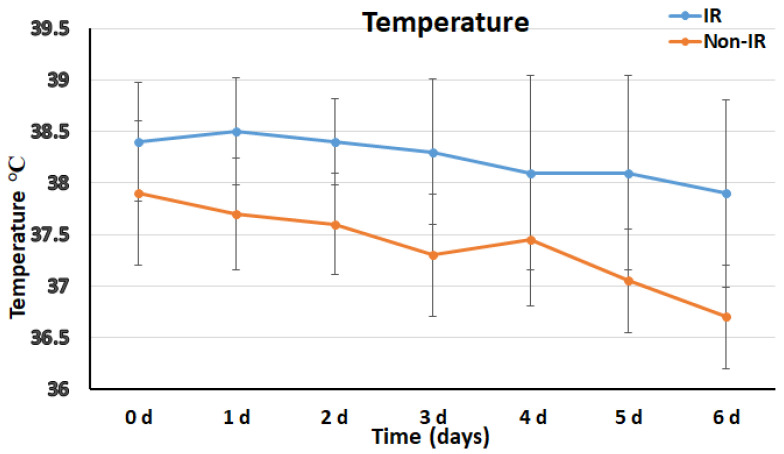
Postoperative distribution and trends of the body temperature in the IR and non-IR group.

Fever in patients with IR showed a later onset if compared with non-IR patients (1.26 ± 0.56 vs. 0.76 ± 0.99 postoperative day, *p* = 0.019) and persisted significantly longer (duration 4.5 ± 1.86 vs. 0.7 ± 0.98 days, *p* < 0.001) (Figure 3). 

**Figure 4:**
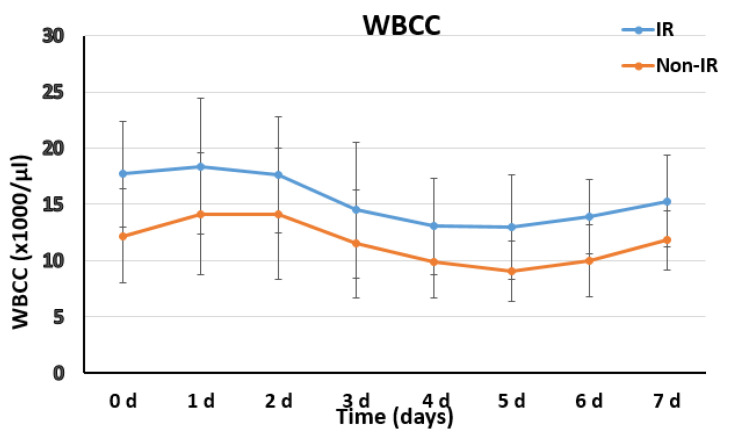
Postoperative distribution and trends of the white blood cell count (WBCC) in the IR and non-IR group.

**Figure 5:**
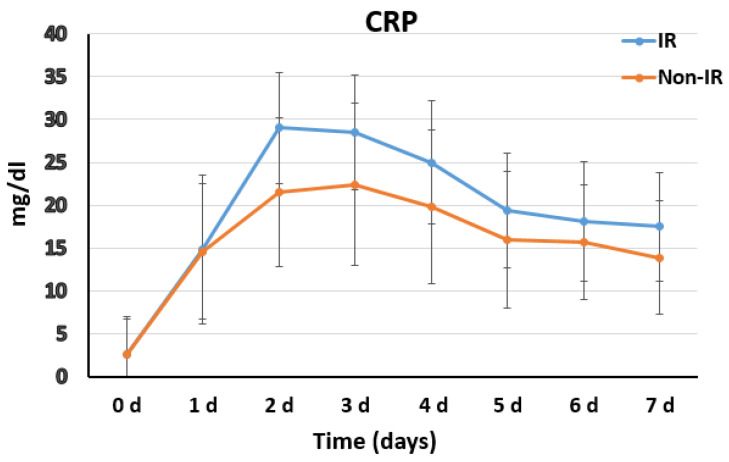
Postoperative distribution and trends of the C-reactive protein (CRP) values in the IR and non-IR group.

With regard to the laboratory data in the IR group, WBCC results significantly increased in the whole first week after surgery with median counts of 14.5 (3. POD), 13 (5. POD) and 15.5 (7.POD) × 1000/µL in the IR group vs. 11.4 (3. POD), 9 (5. POD) and 11.8 (7. POD) × 1000/µL in the non-IR group, corresponding to p of 0.032, 0.002 and 0.014, respectively (Figure 4). C-reactive protein values were tendentially higher in the IR group, resulting in significance in the 3rd postoperative day with 28.4 vs. 22.4 mg/dL, *p* = 0.009 (Figure 5). 

After a multivariate analysis, age, gender, volume of new-onset thrombus and type of aortic pathology consisting of acute dissection, but not procedure (CET/FET) type, remained significantly associated with the occurrence of IR (Table 4).

## 4. Discussion

Systemic stress response and inflammatory activation are known to occur during cardiac surgery such as CET and FET. While in open surgery postoperative IR is classically triggered by surgical trauma due to incision, dissections and tissue manipulation, in the setting of endovascular stent graft implantation, its manifestation can be driven by a sterile inflammation called post-implantation syndrome (PIS). PIS has been originally described in the setting of EVAR for abdominal aortic aneurysm, aiming to elucidate the observed considerable inflammatory response which occurs after endovascular treatment. In the literature, its incidence varies widely. In the setting of type B aortic dissections treated with thoracic endovascular aortic repair (TEVAR), PIS incidence has been previously described amounting to 15.8–31.6% [11,15]; in the context of FET, a previous study reported an incidence of 44.7% [6]. The pathophysiological mechanism of PIS is still largely unknown, while efforts have been made to collect evidence regarding its risk factors. Previous studies described, as predisposing factors for PIS, young age, male gender, implantation of polyester devices and formation of new-onset thrombus.

In the present study, persistent fever without infection focus occurred in the setting of conventional and frozen elephant trunks with an incidence of 33.3%; exclusively in the setting of FET, this manifestation can be classified as PIS. Regarding demographical data, our findings profiled according to the published literature, that IR/PIS patients were significantly younger (53.8 ± 8.9 vs. 60.7 ± 9.6 years, *p* = 0.027) and less frequently of female gender (3.5% vs. 33.3%, *p* = 0.003).

Before the procedures, thrombus volume was not significantly different (9.1 vs. 14 cm^3^, *p* = 0.224). Interestingly, after the procedures this aspect changed, with IR/PIS being significantly associated with the volume of new-onset thrombus (84.4 vs. 3.2 cm^3^, *p* < 0.001). It is known that the hemodynamics in the area of the repair is different after FET and CET, in particular after CET surgery the flow between the graft and the aortic wall can last longer with a consequent impact the thrombus formation.

The role of new-onset thrombus on postoperative inflammatory response was recently effectively investigated by Martinelli et al. [8], comparing the incidence of PIS after endovascular aneurysm sealing (EVAS) and EVAR, with the peculiarity of EVAS consisting of the impediment of new-onset thrombus formation due the endobags occupying the aneurysmatical lumen: their findings confirmed the role of new-onset thrombus as a risk factor for PIS. In the multivariate logistic regression analysis of our cohort, patients with IR appeared to be more frequently affected by acute dissection (*p* = 0.039), and no significant correlation was found with the prothesis type (FET/CET). Regarding the development of new-onset thrombus in our patients, this volume was significantly higher in FET patients compared to CET patients (Figure 2), thus any head-to-head comparison in IR incidence between the procedures would be biased. This result matches with the observation of Inoue et al. [16] on aortic remodeling after treatment for type A aortic dissections, reporting that false lumen thrombosis occurred more frequently with FET than with the CET procedure. Similar results have been observed after treatment for type B acute aortic dissections: a partially thrombosed false lumen was significantly more frequent in the PIS patients when compared with the non-PIS patients [11]. Accordingly, in our multivariate analysis, the volume of new-onset thrombus and type of aortic pathology (acute dissection) were significantly associated with the occurrence of IR (Table 4); the type of procedure (CET vs. FET) was not significantly different in the IR and non-IR groups.

The postoperative systemic inflammatory response and biomarkers characterizing PIS have been previously investigated. In our cohort, we observed a later onset of fever in IR patients, with a longer persistence if compared with non-IR patients (duration 4.5 ± 1.86 vs. 0.7 ± 0.98 days, *p* < 0.001) (Figure 3). In the laboratory data in the IR group, WBCC results were significantly increased in the whole first week after surgery, and C-reactive protein values were tendentially higher (Figure 4 and Figure 5). The finding of higher CRP values in IR patients would support the suggestion to include this parameter in a standardized definition of PIS for FET patients. The role of inflammatory biomarkers as white blood cells (WBC), CRP, interleukins (IL-6, IL-8 and IL-10) and tumor necrosis factor (TNF-α) in PIS have been previously described in the literature [9,10,17,18]. Among the several inflammatory biomarkers involved in the inflammatory cascade, interleukin 6 (IL-6) is the cytokine with the most significant body of evidence linking with PIS. An in vitro study [19] suggested that IL-6 release may be triggered from aneurysmal thrombus formation and cause WBC stimulation and production of TNF-α. Comparing inflammatory biomarkers in a cohort of patients with type B acute aortic dissections, IL-6 was reported to significantly increase within 24 h only in patients who developed PIS [11]. All of these findings support our hypothesis of a role of new-onset thrombus in the pathophysiology of persistent fever without infection focus in CET/FET patients, and our observation of a significant association between new-onset thrombus and IR occurrence, which was confirmed by the multivariate analysis of the here-reported cohort (Table 4). 

In patients undergoing endovascular aneurysm repair with stent grafts differing in material, Voûte et al. [20] observed that stent grafts based on woven polyester were independently associated with a stronger inflammatory response, and in particular the implantation of grafts made of polyester was associated with a higher incidence of PIS compared to expanded polytetrafluorethylene (ePFTE). In our study, we reported patients treated with CET-sealed woven polyester graft or with the FET hybrid prosthesis consisting of a proximal component of sealed woven Dacron (i.e., polyester) and a distal component made of polyester and nitinol stents. Due to the retrospective design of our study, supplemental biomarker examination and an analysis about the role of materials on IR are not possible. Exclusion criteria were designed in order to exclude patients with spurious factors influencing the inflammatory status, in particular, patients with infection focus. Diagnostics in the suspect of infection is well-standardized at our institution; microbiology cultures consisting of the analysis of urine, sputum, stool as well as blood cultures are routinely performed in the presence of fever or elevated laboratory parameters. 

A proinflammatory response may contribute to delayed recovery and morbidity; the influence of PIS on patients’ outcome is, however, unclear. Patients with persistent fever without infection focus incur a relevant risk to undergo numerous diagnostic and therapeutic procedures (e.g., prolonged empirical antibiotic therapy, recurrent chest X-rays) unsuccessfully aiming to identify an infection trigger. In our series, despite comparable operative procedure duration (mean 348.2 ± 95.6 vs. 330.6 ± 65.4 min, *p* = 0.418), IR patients experienced an average additional 3.5 days of prolonged ICU stays in comparison with the non-IR group (9.5 ± 5.8 vs. 6.08 ± 4.7, *p* = 0.033). In the published literature, the data are controversial: some authors reported longer hospitalization stays in PIS patients after EVAR [10,21] without significant differences in mortality [21]; others, in the context of TEVAR for type B acute aortic syndromes, detected no impact in the in-hospital outcome [11,15], but increased rates of major adverse events (excluding all-cause mortality) at a mean follow-up of 4 years [11].

In summary, with the growing application of hybrid stent grafts such as FET for aortic arch lesions and for extensive thoracoabdominal aortic pathologies, postoperative persistent inflammatory responses in the form of a sterile fever and PIS are becoming increasingly relevant in postoperative care. The pathophysiological mechanisms of IR and PIS are still largely unknown. Various risk factors have been postulated, and IR and PIS incidence may be influenced by graft materials and triggered by the formation of new-onset aortic thrombus.

To date, it remains uncertain if IR affects long-term clinical outcomes and if patients warrant closer surveillance. Prospective studies investigating the underlying etiology and evaluating treatment options and their effect on clinical outcome are needed.

This study presents several limitations. First, our investigation is based on retrospective data; secondly, we do not routinely assess inflammatory biomarkers (e.g., IL-6), which could have helped to better differentiate clinical manifestations of PIS, which may overlap with the systemic inflammatory response by surgical trauma. Lastly, our cohort is small, due to the single-center design. Further research is necessary to better understand the impact, risk factors and biological mechanisms supporting persistent inflammatory response and post-implantation syndrome, and the role of thrombus in their etiology.

## 5. Conclusions

In the context of CET and FET, persistent inflammatory response was not rare, occurring in 33.3% of the patients with persistent fever without infection focus. Persistent inflammatory response after CET, and PIS after FET are associated with a higher volume of new-onset thrombus and significantly prolonged ICU stays. Further studies to validate these observations and investigate the prognostic impact and therapeutic options are needed.

## Figures and Tables

**Table 1:** Baseline characteristics, comorbidity and medication treatment at admission of the study population divided according to the presence of IR.

	Total (n = 57)	IR (n = 19)	Non-IR (n = 38)	*p*-Value
Demographic characteristics				
Age (years) mean ± SD	58.4 ± 12.6	53.8 ± 8.9	60.7 ± 9.6	**0.027**
Age < 60 years, n (%)	27 (47.3)	14 (73.6)	13 (34.2)	**0.005**
Female gender, n (%)	21 (36.8)	2 (3.5)	19 (33.3)	**0.003**
Type of aortic pathology				
Acute dissection	32 (56.1)	10 (52.6)	22 (57.8)	
Post dissection aneurysm	11 (19.2)	8 (42.1)	3 (7.8)	
Aneurysm	14 (24.5)	1 (5.2)	13 (34.2)	**0.002**
Max. diameter	65.7 ± 2.5	63.3 ± 4	64.4 ± 3.2	0.690
Medical history, n (%)				
Hypertension	45 (78.9)	15 (78.9)	30 (78.9)	0.626
Previous stroke/TIA	6 (10.5)	1 (5.2)	5 (13.1)	0.652
COPD	8 (14)	4 (21)	4 (10.5)	0.420
Coronary artery disease	9 (15.7)	5 (26.3)	4 (10.5)	0.143
Chronic heart disease	10 (17.5)	3 (5.2)	7 (18.4)	0.650
Current/previous smoker	24 (42.1)	8 (42.1)	16 (42.1)	0.530
Atrial fibrillation	9 (15.7)	2 (10.5)	7 (18.4)	0.361
Medication treatment, n (%)				
ß-Blocker	31 (54.3)	10 (5.2)	21 (55.2)	0.806
ACEIs	15 (26.3)	6 (31.5)	9 (23.6)	0.451
Aspirin	6 (10.5)	0 (0)	6 (15.7)	0.162
Statins	10 (17.5)	5 (26.3)	5 (13.1)	0.275

ACEIs, angiotensin-converting enzyme inhibitors; COPD, chronic obstructive pulmonary disease; TIA, transient ischemic attack; SD, standard deviation; statistically significant *p*-values are marked in bold.

**Table 2:** Procedural and perioperative data of the study population overall and in the subgroup distributed according to the presence of IR.

	Total (n = 57)	IR (n = 19)	Non-IR (n = 38)	*p*-Value
Procedure characteristics (mean ± SD)				
OP duration (minutes)	336.4 ± 76.4	348.2 ± 95.6	330.6 ± 65.4	0.418
ICU stay (days)	7.2 ± 5.3	9.5 ± 5.8	6.08 ± 4.7	**0.033**
In-hospital length of stay (days)	14.7 ± 5.9	15.6 ± 4.9	14.3 ± 6.3	0.377
Prosthesis type, n (%)				
Frozen elephant trunk	38 (66.6)	17 (44.7)	21 (55.2)	
Conventional elephant trunk	19 (33.3)	2 (10.5)	17 (89.4)	**0.009**
Thrombus volume measurement, median (range), n (%)				
Preoperative mural thrombus (cm^3^)	10.1 (2–408)	9.1 (3–340)	14 (2–408)	0.224
Postoperative new-onset thrombus (cm^3^)	9.7 (0.2–376)	84.4 (2–376)	3.2 (0.7–68)	**<0.001**

ICU, intensive care unit; statistically significant *p*-values are marked in bold.

**Table 3:** Postoperative inflammatory markers in the whole population and divided according to the presence of IR.

	Total (n = 57)	IR (n = 19)	Non-IR (n = 38)	*p*-Value
Body temperature				
Fever	42 (73.6)	19 (100)	23 (60.5)	
Fever onset (POD)	0.93 ± 0.90	1,26 ± 0.56	0.76 ± 0.99	**0.019**
Fever duration, day	2 ± 2.26	4.5 ± 1.86	0.7 ± 0.98	**<0.001**
3. POD				
WBCC (×1000/µL), median (IQR)	12.5 (9–17.2)	14.5 (11.5–19.2)	11.4 (8.5–15.1)	**0.032**
CRP (mg/dL), median (IQR)	25.3 (18.9–29.7)	28.4 (23.9–31.6)	22.4 (14.7–28)	**0.009**
5. POD				
WBCC (×1000/µL), median (IQR)	9.8 (7.6–12.4)	13 (9.5–15.8)	9 (7–11.5)	**0.002**
CRP (mg/dL), median (IQR)	19 (11.9–21.3)	19.7 (18.9–23.3)	16 (8.9–21.4)	0.095
7. POD				
WBCC (×100/µL), median (IQR)	12.8 (10.4–14.8)	15.3 (12.2–16.6)	11.8 (9.7–13.6)	**0.014**
CRP (mg/dL), median (IQR)	15.4 (10.5–19)	17.5 (10.7–23)	13.9 (10–18)	0.129

CRP, C-reactive protein; WBCC, white blood cell count; POD, postoperative day; IQR, interquartile range; PCT, procalcitonin; statistically significant *p*-values are marked in bold.

**Table 4:** Multivariate analysis of the significant variables associated with the occurrence of IR.

	Univariable Analysis			Multivariate Logistic Regression		
	OR	95% CI	*p*-Value	OR	95% CI	*p*-Value
Age < 60 years	5.38	1.58–18.26	**0.007**	0.748	0.561–0.996	**0.044**
Volume of new-onset thrombus	1.057	1.020–1.094	**0.002**	1.1	1.01–1.23	**0.025**
Femal gender	0.118	0.024–0.581	**0.009**	0.0001	0.0001–0.312	**0.038**
Prosthesis type	0.145	0.029–0.719	**0.018**	12.7	0.106–1530	0.100
Type of aortic pathology	1.87	0.928–3.77	0.080	746	1.42–3923	**0.048**
Aneurysm	0.077	2.56–0.91	**0.035**			
Acute dissection	0.455	1.68–0.12	**0.039**			
Post-dissection aneurysm	2.66	0.18–2.30	0.147			

CI: confidence interval; OR: odds ratio; statistically significant *p*-values are marked in bold.

## Data Availability

Not applicable.
